# Comparison of Threshold and Tolerance Nociceptive Withdrawal Reflexes in Horses

**DOI:** 10.3390/ani11123380

**Published:** 2021-11-26

**Authors:** Selina Mühlemann, Massimo Leandri, Åse Ingvild Risberg, Claudia Spadavecchia

**Affiliations:** 1 Department of Clinical Veterinary Medicine, Anaesthesia Section, Vetsuisse Faculty Bern, 3012 Bern, Switzerland; muehlemann.selina@gmail.com; 2 Department of Neuroscience, University of Genova, 16132 Genova, Italy; massimo.leandri@unige.it; 3 Faculty of Veterinary Medicine, Norwegian University of Life Sciences, 1433 Ås, Norway; aase.risberg@nmbu.no

**Keywords:** horse, equine, nociceptive withdrawal reflex, nociception, tolerance, electromyography

## Abstract

**Simple Summary:**

Nociception is the physiological basis of the complex experience of pain. An established model for its quantification in equine studies is based on the nociceptive withdrawal reflex evoked by electrical stimulation of a sensory nerve. The reflex is recorded via electromyography and it is common to determine the threshold at which a nociceptive-specific reflex activity can be observed. In the present study, the classical methodology was expanded for a deeper understanding of the physiology of nociceptive reflexes in horses. First, for each individual horse, a threshold was determined as the minimal stimulation intensity able to evoke a nociceptive withdrawal reflex. Second, the stimulation intensity was stepwise increased up to tolerance, which was defined as the stimulus that is able to elicit the maximal tolerable behavioral reaction. The characteristics of the reflex activity on the electromyographic records were compared for threshold and tolerance stimulation intensities. At tolerance, the reflex became faster and wider than at threshold, indicating that either a spinal summation mechanism or the recruitment of faster sensory fibers occurs in response to high-intensity noxious stimuli. A novel endpoint (i.e., tolerance) can now be considered when applying the nociceptive withdrawal reflex model in equine studies.

**Abstract:**

The nociceptive withdrawal reflex (NWR) is used to investigate nociception in horses. The NWR threshold is a classical model endpoint. The aims of this study were to determine NWR tolerance and to compare threshold and tolerance reflexes in horses. In 12 horses, the NWR was evoked through electrical stimulation of the digital nerve and recorded via electromyography from the deltoid. Behavioral reactions were scored from 0 to 5 (tolerance). First, the individual NWR threshold was defined, then stimulation intensity was increased to tolerance. The median NWR threshold was 7.0 mA, whereas NWR tolerance was 10.7 mA. Upon visual inspection of the records, two main reflex components R1 (median latency 44 ms) and R2 (median latency 81 ms) were identified at threshold. Increasing stimulation intensity to tolerance led to a significant increase in the amplitude and duration of R1 and R2, whereas their latency decreased. At tolerance, a single burst of early, high-amplitude reflex activity, with a median latency of 39 ms, was detected in 15 out of 23 stimulations (65%). The results of this study suggest that (1) it is feasible to determine NWR tolerance in horses and (2) high-intensity stimuli initiate ultrafast bursts of reflex activity, which is well known in practice and has now been quantified using the NWR model.

## 1. Introduction

In the past twenty years, the nociceptive withdrawal reflex (NWR) has been widely used as a model in equine pain research [1,2,3,4,5,6,7,8,9,10,11,12]. First described by Sherrington [13], the NWR is a physiological, polysynaptic spinal reflex protecting the body’s integrity against an external, noxious stimulus [14,15]. The NWR is a complex, adaptable response involving several muscle groups, elicited when the stimulation intensity is sufficient to depolarize thinly myelinated nociceptive Aδ fibers, with the purpose of generating an appropriate defensive withdrawal reaction [16]. In experimental settings, the NWR is usually evoked through transcutaneous electrical stimulation of a limb peripheral nerve or via cutaneous electrical stimulation [8,15,17]. The NWR threshold is generally defined as the minimal stimulation intensity needed to evoke a stable reflex [7,8], which can be quantified using electromyography recorded from a muscle involved in the withdrawal reaction. Several studies reported a strong correlation between the NWR threshold and perceived pain in humans [17,18,19]. Based on these findings, numerous pharmacological trials on analgesics and anesthetics have been carried out using the NWR model in humans [17,20,21] and horses [1,2,3,5,22,23]. Furthermore, the NWR model can provide insights into the physiology of dynamic nociceptive processes [24]. As an example, the phenomenon of temporal summation (a single non-noxious stimulus, unable to evoke the NWR when given alone, can be integrated, causing pain and facilitation of the NWR if given repeatedly) can easily be evoked and quantified using the NWR [25,26,27]. Furthermore, by progressively increasing the stimulation intensity above a threshold value, a stimulus–response curve can be drawn [3,7]. The curve shape provides information about the system gain, adding a dynamic dimension to the simple static measure of the threshold [26].

In horses, it has been shown that it is possible to elicit and record reliable nociceptive reflexes [28]. In previous studies evaluating stimulus–response curves, the maximal stimulation intensity applied corresponded to 1.3 times the threshold [3,7]. Individual differences in behavioral responses to increasing stimulation intensities were noticed, indicating that the final endpoint was potentially not truly comparable among individuals. Increasing the stimulation intensity further would possibly allow one to determine the individual tolerance NWR, defined as the reflex evoked by the highest stimulation intensity judged to be tolerable for a certain subject. Thus, a more comparable endpoint would be obtained. By evaluating the differential characteristics of threshold and tolerance NWR, new insight into the mechanisms of nociceptive recruitment could be gained, contributing to a better understanding of species-specific behavioral reactions to noxious inputs in real life. Moreover, in the context of pharmacological investigations, having two different endpoints allows us to draw more comprehensive and informative conclusions about drugs’ modes of action. Indeed, a certain drug could be merely effective in reducing tolerance but not threshold: such a finding would indicate that the drug does not alter the basic nociceptive processing, yet it is able to smooth the perceived pain intensity for high-level stimuli. This situation is potentially very interesting in clinical settings, in which there might be no need for a total ablation of nociception, but rather a modulation of pain peaks.

The purposes of the study reported here were to evaluate the feasibility of determining the tolerance NWR in standing unmedicated horses and to describe and compare the neurophysiological characteristics of the reflexes evoked by stimulations at the threshold and tolerance levels. It was hypothesized that it would be feasible to determine a consistent tolerance NWR in standing horses and that this tolerance NWR would be reliably characterized by shorter latency and higher amplitude than the threshold NWR.

## 2. Materials and Methods

Twelve adult Swiss Warmblood geldings (mean weight 602 kg, SD ± 39 kg; mean age 15.4 years, SD ± 4.5 years) were included in this study, which was part of a larger crossover pharmacological trial investigating the antinociceptive effects of lidocaine and dexmedetomidine [1]. The data reported in the present study were collected during baseline measurements obtained in a pilot trial (2 horses) and in the main trial (11 horses) before drug administration. Only one horse was included both in the pilot and in the main trial. Of the 11 horses included in the main crossover trial, 10 underwent two experimental sessions at least 2 weeks apart. One horse became nervous during the pharmacological trial following baseline measurements and was excluded from the second experimental session. All horses were judged to be clinically healthy throughout the experimental period. Experiments were approved by the Cantonal Committee of Animal Experimentation, Bern, Switzerland (BE 67/10).

In the afternoon prior to the experimental day, horses were familiarized with the experimental room. During the experiment, horses were restrained in a stock to which they were already acquainted for routine procedures. Hair was clipped over the right lateral palmar digital nerve, over the back and over the deltoid muscle to prepare the electrode sites. On the day of the experiment, horses were lunged for 30–45 min. The total duration of the experiment, including the pharmacological trial, was approximately 3–4 h, during which the horses were restrained in stocks. The skin at the electrode sites was cleaned and degreased. For electrical stimulation, two self-adhesive electrodes (Neuroline 700, Ambu, Ballerup, Denmark) were applied over the right digital nerve. A minimal distance of 20 mm was maintained between the two electrodes. A ground electrode was placed on the horse’s back. A pair of self-adhesive electrodes (Synapse, Ambu, Ballerup, Denmark) were placed over the right deltoid muscle in order to record surface electromyograms (bipolar recording). The electrodes were placed parallel to the muscle fibers, 20 mm apart. Impedance was measured using a multimeter (113 True RMS multimeter, Fluke, Everett, WA, USA) before each experimental session and maintained at <3 kΩ. Flexible leads were connected to the electrodes; electrodes and leads were secured with adhesive bandages to avoid displacement.

Electrical stimulation and recording were performed with a purpose-built computerized system [8]. One stimulus consisted of a train of five, 1-millisecond, constant-current, square wave pulses, delivered at a frequency of 200 Hz [29]. For each stimulation, electromyographic activity was recorded from 100 ms prior to until 400 ms after the stimulus; the total recording time was 500 ms, with a sampling frequency of 1 kHz. After electrode placement, spontaneous electromyographic activity was recorded from the deltoid muscle to ensure the correct functioning of the system. In order to acquaint the horse to the sensation evoked by an electrical stimulus, stimulation was started at 1 mA and gradually increased in 1 mA steps until a reaction score of 1 (Table 1) was achieved. The stimulator was activated manually when the horse was standing quietly without movement of the head or limb and with the weight distributed equally to all four limbs. Recordings were discarded when they coincided with any activity of the horse. There was no need for human contact during the experimental procedure and the horse could not link the stimulation to any external action or noise. The time elapsing between successive stimulations was randomized and ranged between 10 and 90 s to prevent reflex habituation, as previously recommended [30,31].

The experimental session was started at a stimulation intensity of 1 mA, and then increased by steps of 1 mA, until a NWR could be recognized on the EMG, accompanied by a minimal reaction score of 2 (Table 1). Stimulation at the same intensity was repeated in order to check the reproducibility of the response. If a certain stimulation intensity was able to evoke an NWR and reaction score of 2 twice, that intensity was defined as the NWR threshold. If not, stimulation intensity was adjusted in steps of 0.1–0.5 mA until the NWR threshold could be defined. Stimulation was then continued with intensities starting at 0.9 times the NWR threshold, increased in steps of 10% of the NWR threshold until a score 5 behavioral response (vigorous whole-body reaction with immediate lifting of the limb; Table 1) in the presence of a clearly recognizable NWR on the EMG was observed. At that point, stimulations were terminated, and the highest stimulus intensity (mA) reached was defined as NWR tolerance.

The EMG record was visually inspected to detect the presence of one or more reflex components following the onset of stimulation. Latency of a reflex component was defined as the time elapsed between the onset of the stimulus and the onset of the specific reflex deflection from baseline on the EMG record. To quantify the reflex response, the root-mean-square (RMS) amplitude was calculated from the onset to the offset of the reflex component under evaluation. The RMS of the 100 milliseconds preceding the stimulation was also calculated and considered as the background EMG activity in the resting state. The relative amplitude (from now on simply named the amplitude) of each detected reflex component was calculated as the ratio between the specific reflex RMS and the RMS amplitude of the background activity.

Threshold intensity was defined as the minimum stimulus intensity able to evoke an NWR response of at least 3 times the background activity. In addition, the main EMG burst had to last at least 10 milliseconds and had to be followed by a reaction scored ≥2. Tolerance was defined as the stimulation intensity accompanied by a reaction score of 5 (vigorous whole-body reaction and immediate lifting of the limb) during the NWR recruitment [8,28].

Behavioral reactions following each stimulation were directly observed and scored by two investigators (Å.I.R. and C.S.) using a system modified from Rohrbach et al. [22] according to Table 1. Scores attributed to each reaction by the 2 observers were briefly compared and the agreed score was added to the respective EMG recording. If there was disagreement between observers or if one of the observers was unable to attribute a score, the stimulation was repeated at the same intensity.

Based on tests for normality and sample size, nonparametric statistical tests were chosen. Results are presented as median and interquartile ranges (IQR; 25% to 75% interquartile range). A Wilcoxon signed rank test was used to evaluate whether there was a significant difference between the first and the second experimental session, for several variables (i.e., reaction score, reflex latency and amplitude at both the threshold and tolerance levels). The same test was used to detect differences in reflex characteristics for recordings obtained at threshold and tolerance levels. The Friedman test was used to compare the same variables at increasing stimulation intensities during recruitment (i.e., 0.9, 1.0, 1.1, 1.2 and 1.3 times the NWR threshold). When needed, the post hoc Tukey Kramer test was applied for multiple comparisons. Correlation among behavioral scores and the intensity of stimulation was calculated with a Spearman rank test. Interindividual differences among horses were analyzed with the Kruskal–Wallis test. Overall significance was set at a value of *p* < 0.05. Data were analyzed using dedicated software [32,33].

## 3. Results

The NWR could be evoked in all 12 horses and recruitment up to tolerance was feasible in all horses. For 10 horses (one pilot horse and nine horses included in the main pharmacological trial), two baseline measurements were available. The median stimulation intensity required to evoke an NWR (NWR threshold) was 7.0 mA (IQR, 6.0–8.3 mA), whereas the median stimulation intensity required to evoke NWR tolerance was 10.75 mA (IQR, 8.3–12.4 mA). Tolerance was reached at a median of 1.4 times NWR threshold (IQR 1.3–1.5 times NWR threshold). Stimulation intensities (mA) needed to evoke the NWR threshold and tolerance were not significantly different among horses or between the two experimental sessions.

In order to evaluate the repeatability of the responses, reflex characteristics (latency, amplitude and duration) at both threshold and tolerance stimulation levels were compared for the two experimental sessions. As differences between the two experimental sessions were not detected, the data obtained during the pilot trial and the two experimental sessions were pooled together for the neurophysiological characterization of the reflexes (Table 2 and Table 3).

**Figure 1:**
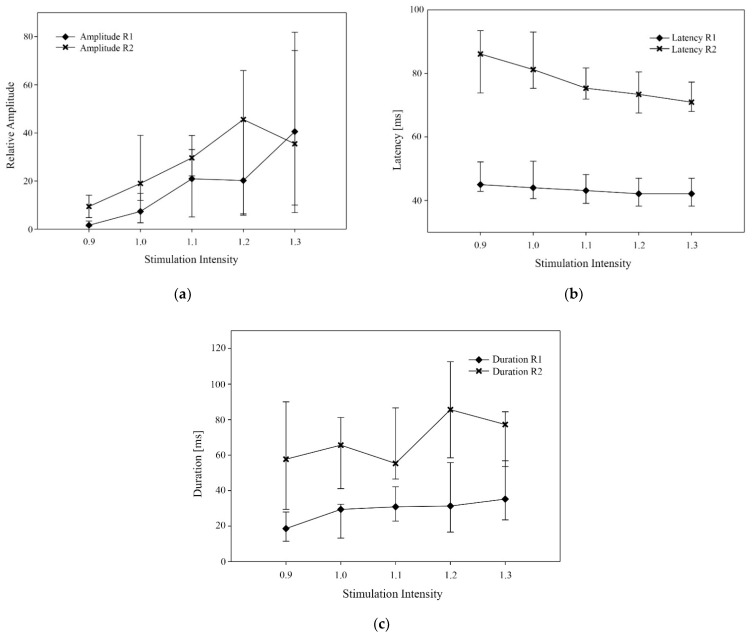
Median value (and interquartile range) of different variables for R1 and R2 at different stimulation intensities (0.9, 1.0, 1.1, 1.2 and 1.3 times the nociceptive withdrawal reflex threshold value). (**a**) relative amplitude, (**b**) onset latency (ms), (**c**) reflex duration (ms).

The data are summarized in Table 2 and Table 3. Increasing stimulation intensity from threshold to tolerance led to a significant increase in the amplitude and duration of both R1 and R2 (Figure 1a,c). The latency of both reflex components decreased for increasing stimulation intensities (Figure 1b). A positive correlation between behavioral reaction score and stimulation intensity was found (r = 0.75; *p* < 0.0001).

**Figure 2:**
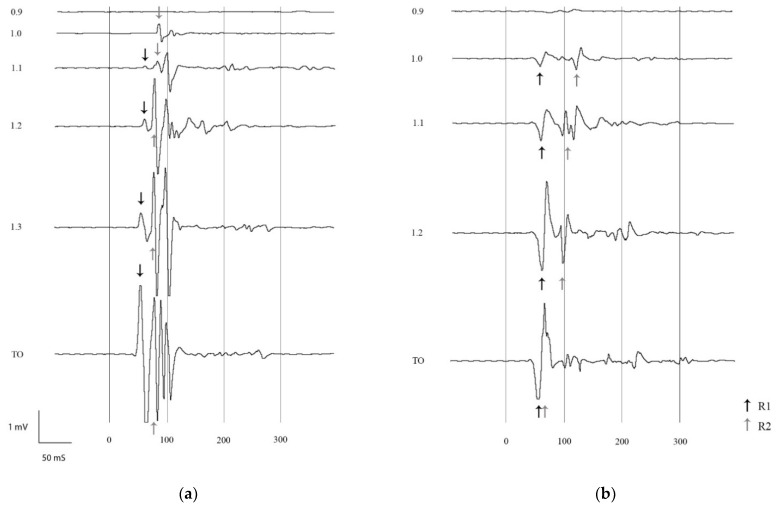
Electromyograms recorded from the right deltoid muscle after electrical stimulation of the right lateral palmar digital nerve in two horses (**a**,**b**). Stimulation intensity ranged from 0.9 times NWR threshold to NWR tolerance (TO), a stimulation accompanied by a reaction score of 5. Stimulation intensity was increased in steps of 10%. In the illustrations, the *x*-axis represents time (ms) and the *y*-axis represents amplitude (mV). Stimulation onset was at time 0. Where present, R1 is marked with black arrows and R2 with gray arrows. (**a**) The deflection from baseline seen at NWR threshold is R2. In the recording at 1.1 times NWR threshold R1 is preceding R2. R1 and R2 can be distinguished in all recordings, but they become closer for increased stimulation intensity, due to a shortening in the latency of R2. (**b**) At NWR threshold, R1 and R2 can be distinguished from each other, whereas at NWR tolerance only one reflex burst can be observed.

The R1 component was detected in 13 of 23 baseline recordings at threshold and in 22 of 23 recordings at tolerance. The R2 component was detected in 22 of 23 stimulations at threshold and in only 8 of 23 recordings at tolerance. Indeed, in several EMG recordings, a single burst of reflex activity, that seemed to originate from R1 and R2 overlapping, was detected in the R1 time window (Figure 2b). This was most frequent at higher stimulation intensities. At threshold, a single reflex burst could be observed only in 1 of 23 EMG recordings, whereas at tolerance it was observed in 15 of 23.

Individual differences were observed among horses. Out of 12 horses, two showed no R1 and R2 overlapping at any stimulation intensity. In two horses, R2 appeared to get closer to R1 for higher stimulation intensities; however, complete overlapping was not observed (Figure 2a). In the remaining eight horses, no overlapping was seen at threshold, whereas it appeared at higher stimulation intensities (Figure 2b). In recordings preceding the first overlapping, the latency of R2 was decreasing and getting closer to R1. Interestingly, in horses undergoing two experimental sessions, this individual pattern was maintained. Similarly, reaction scores showed good repeatability for individual horses. At both threshold and tolerance stimulation levels, no significant differences in reflex characteristics (latency, amplitude or duration) were present among horses.

## 4. Discussion

The results of this study indicate that it is possible to determine not only the NWR threshold, but also the NWR tolerance in conscious standing horses. As expected, increasing stimulation intensities exerted considerable modulation of both the behavioral pattern and the electromyographic characteristics of the evoked responses. Although the NWR threshold is a well-established endpoint for the NWR model in horses [28], NWR tolerance has never been characterized in detail to date. Tolerance was defined as the maximal stimulation intensity judged to be acceptable by an individual horse restrained in stocks. Two investigators (Å.I.R. and C.S.) applied an ad hoc scoring system to evaluate the stimulation-evoked behavioral reactions and to determine tolerance. Interestingly, they always agreed on attributing a score of five to a certain response, indicating that despite the potential subjectivity of the judgement, the tolerance endpoint is easily recognized and can be proposed in a reflex recruitment protocol. The pharmacological study of which the present data served as the baseline measurements represents the first application of tolerance as a new endpoint of nociceptive testing in horses [1].

It was hypothesized that tolerance NWR would be reliably characterized by shorter latency and higher amplitude than threshold NWR. Accordingly, we observed that stimulation intensities at tolerance level elicited reflexes were characterized by higher amplitude and shorter latency than at threshold. These findings are in agreement with various previous studies in horses [7,8] and other domestic species [34,35]. In the equine studies, the 400-millisecond interval after stimulation was usually divided into three epochs (0–80, 80–250 and 250–400 ms after stimulation) to quantify the NWR and other reflex components. These epochs were initially selected on the basis of nerve conduction velocity data obtained from human and previous experiments in horses [8,36,37,38,39,40]. In the present study a different approach was chosen to better describe the reflex characteristics and their modifications occurring during the recruitment process: through a visual inspection of the EMG recordings, two separate reflex components were identified. They were arbitrarily named R1 and R2, according to the order of their appearance after stimulation. A similar nomenclature is classically used to describe the blink reflex in humans and horses [9,41].

The reflex termed R2 in the present investigation corresponds to the classically described NWR, as it appears at the threshold stimulation level in the epoch 80–250 ms after stimulation onset [28]. As previously described in equine NWR recruitment trials, R2’s amplitude and duration increased, whereas its latency decreased for higher stimulation intensities [7,8]. However, at stimulation intensities approaching tolerance, R2 was often not distinguishable from R1. Indeed, a single burst of high-amplitude reflex activity that seemed to originate from the overlapping of R1 and R2 was detected earlier than 80 ms after stimulation onset, in the R1 time window.

The amplitude of both R1 and R2 showed considerable variability, as commonly observed for polysynaptic reflexes due to its susceptibility to supraspinal modulation [42]. The variable with the narrowest interquartile ranges was latency, which is also to be expected, as latency is dependent on neuronal conduction velocities and on the stimulation level, which are not sensitive to individual differences or potential environmental influences [42]. R1 was not present in all EMG recordings, but it appeared significantly more often at tolerance than at threshold stimulation intensity. Not only did R1 appear more likely at tolerance than at threshold, but it also showed increased amplitude, shorter latency and longer duration. Remarkably, the increase in amplitude of R1 was more pronounced than the increase in amplitude of R2, classically considered as NWR (a nine-fold increase in the median relative amplitude for R1 from threshold to tolerance, as opposed to a four-fold increase for R2) [28]. Conventionally, reflex activity within the 80-millisecond epoch following stimulation is considered to be conducted by Aβ fibers with a conduction velocity of 50 to 60 m/s [8,37,38,39]. The observed R1 amplitude increase and shortening of latency during recruitment is a feature thought to be associated with reflex activity of nociceptive origin [15,18]. Electrically-evoked nociceptive reflexes are commonly attributed to Aδ fibers, characterized by a conduction velocity in the range of 4 to 36 m/s [39]. With an afferent pathway of approximately 1.6 m length, an afferent latency of at least 45 ms has to be assumed. Adding a mean efferent time of 10 ms, accounting for synaptic delay [15,43,44,45] and motor component [15,46], a minimal latency of 55 ms should be expected for Aδ-fiber-related reflex activity. At threshold, R2 appeared indeed after 55 ms. At tolerance, however, most horses showed an early pronounced reflex component (<50 ms latency) and no distinguishable activity burst in the classical NWR epoch (80–250 ms). Additionally, all horses reacted with a strong whole-body reaction and immediate lifting of the limb when stimulated at the tolerance level. Previous work on NWR has shown that increasing stimulation intensity leads to an increase in the perceived pain and to an increased amplitude of the elicited reflex in both humans and animals [7,8,17,18,19,34,35]. The reflex characteristics observed at tolerance strongly suggest that nociceptive reflexes can begin as early as 35 ms after stimulation onset. This early reflex activity could be explained by the recruitment of fast conducting Aβ fibers for high-intensity stimulations. In the same direction, an important role of Aβ fibers in nociceptive processing has been previously reported for mice, rats, guinea pigs, cats and monkeys [47]. Recently, an extensive investigation in humans showed similar results [48]. On the other hand, the involvement of Aβ fibers at higher stimulation intensities in our experiment does not fit with their chronaxie, which is lower than for slow conducting fibers. The reasons for such paradoxical results have not been investigated in our work, but one hypothesis could be that stronger stimuli could recruit larger number of fibers, yielding a spatial summation at the reflex center.

This last hypothesis is seemingly the most probable. Indeed, in classical human and animal NWR studies, the standard stimulus used consists of a train of five pulses, which has the basic purpose of eliciting central temporal summation, essential for the reflex and its cortical perception. Although no specific equine data are available in this regard, a previous experiment in dogs confirmed that a single pulse is much less effective than a train in eliciting nociceptive reflexes [34]. In real life, immediate and targeted responses to painful stimuli are well known to be part of the typical behavioral repertoire of horses.

The EMG reflex pattern and threshold of the NWR can vary among body regions, depending on the biological function of the reflex [15,49]. In the present study the NWR was recorded from the deltoid muscle. The NWR threshold recorded from this muscle was higher than those previously reported for other muscles involved in withdrawal reactions in response to stimulation of the digital nerve. In a previous study in ponies, recordings from the common digital extensor and the deltoid muscle were performed contemporaneously [4]. The deltoid showed higher NWR thresholds than the common digital extensor. Interestingly, anesthetised ponies never moved the stimulated limb at stimulation intensities that were able to evoke an EMG reflex response in the common digital extensor, but there was always visible flexion-protraction at stimulation intensities able to evoke the NWR in the deltoid muscle. Another possible explanation for the higher threshold found in this study is linked to the fact that a minimum reaction score of two (a moderate brisk whole-body reaction) has been added as a condition for defining the NWR threshold. This was carried out to make the determination more consistent and repeatable and to ensure a minimal degree of unpleasantness for the perceived stimulus. In the majority of the previous reports, threshold determination was based purely on the observed EMG record. In that case, the reaction score accompanying the threshold was most frequently one, a slight brisk whole-body reaction. Accepting a slight brisk reaction as the threshold reflex was criticized by peers as potentially insufficient to indicate nociception.

It would be interesting to investigate whether the observed reflex pattern at tolerance is dependent on the stimulation site and what similar stimulations in other species would reveal. From an evolutionary perspective it might be assumed that in prey animals, nocifensive behavioral patterns and their neurophysiological correlates are further developed than in predators.

A methodological limitation of the current study is the fact that limb lifting, occurring at the tolerance stimulation level simultaneously with the main burst of EMG reflex activity, was only visually observed but not objectively quantified. An accelerometer or an electrical goniometer should have been added to characterize the extent of limb withdrawal, thus confirming the exact timing of the behavioral reaction in relation to the EMG deflection.

Furthermore, stimulation was applied transcutaneously via surface electrodes positioned over the nerve trunk. The purpose of this was to depolarize the nerve directly, bypassing cutaneous nociceptors. Whether the nerve trunk was truly activated already at the lowest stimulation intensities cannot be confirmed by the present experiment. A way to verify this hypothesis could consist in the investigation of reflexes evoked by pure skin stimulation, by applying electrodes away from the nerve. If reflex characteristics did not differ, it would mean that the intended nerve stimulation of the present study was rather a cutaneous stimulation at low intensities. Another method that could be used to shed light on the exact sequence of nerve fiber activation evoked under the present experimental setting would consist in applying neurography. As recording needle electrodes need to be inserted in close proximity to the stimulated sensory nerve, it is not realistic to perform this test in awake unmedicated horses.

Only 12 geldings were included in the present study. Although, based on past experience, we do not expect important sex-related differences to be present in terms of the NWR threshold, it would be necessary to specifically evaluate this point to exclude any potential sex-related influence on these findings. The methodology applied in the present study for the evaluation of the EMG record, based on the identification of the R1 and R2 reflex component, has been proven to be effective and adequate for describing the NWR, even if differences among horses have been detected. For future investigations intending to apply NWR tolerance to horses or other species, we suggest considering a similar approach. Following the temporal and spatial evolution of the NWR’s main components over a range of stimulation intensities might allow the definition of intensity-dependent, species-specific windows of interest that can then be applied in future pharmacological trials.

## 5. Conclusions

The results of this study suggest that (1) it is possible to determine not only the NWR threshold, but also the NWR tolerance in horses and (2) high-intensity noxious stimuli initiate ultra-fast bursts of reflex activity, accompanied by immediate gross withdrawal reactions, which belong to the typical behavioral repertoire of this species and can now be precisely characterized with the NWR model.

## Figures and Tables

**Table 1:** Scoring system for behavioral reactions during stimulation (modified from Rohrbach et al., 2009 [22]).

Score	Behavioral Reaction
0	No reaction
1	Slight brisk whole-body reaction
2	Moderate brisk whole-body reaction
3	Prompt whole-body reaction and delayed lifting of the stimulated limb
4	Immediate lifting of the stimulated limb
5	Vigorous whole-body reaction with immediate lifting of the stimulated limb

**Table 2:** Median (interquartile range) values obtained during electromyographic recordings of the right deltoid muscle in 12 horses stimulated over the right lateral palmar digital nerve with 25-ms train-of-5 constant-current pulses (duration 1 ms) delivered by a computer-controlled stimulator at various stimulation intensities: 0.9, 1.0, 1.1, 1.2 and 1.3 times the nociceptive withdrawal reflex (NWR) threshold (TH).

	Stimulation Intensity ^1^					
Variable	0.9	1.0 (TH)	1.1	1.2	1.3	P^2^
Reaction Score	1 (1–2)	2 (2–3)	2 (2–3)	3 (3–4)	4 (4–5)	
Relative Amplitude R1	1.6 (1.2–3.4) ^a^	7.3 (2.6–14.8) ^a,b^	20.9 (5.1–33.1) ^b,c^	20.2 (6.3–45.1) ^b,c^	40.6 (10–74.3) ^c^	<0.001
Relative Amplitude R2	9.4 (4.8–14-1) ^a^	19 (11.9–39) ^a,b^	29.6 (22.1–39) ^a,b^	45.6 (5.8–65.9) ^b^	35.5 (6.9–81.9) ^b^	<0.01
Latency R1 (ms)	45 (43–52) ^a^	44 (41–52) ^a,b^	43 (39–48) ^b,c^	42 (38–47) ^c^	42 (38–47) ^c^	<0.001
Latency R2 (ms)	86 (74–94) ^a^	81 (75–93) ^a^	75 (72–82) ^a,b^	73 (68–81) ^a,b^	71 (68–77) ^b^	<0.001
Duration R1 (ms)	19 (12–28) ^a^	29 (13–32) ^a,b^	31 (23–42) ^a,b^	31 (17–56) ^b^	35 (24–57) ^b^	<0.001
Duration R2 (ms)	58 (29–90) ^a^	66 (41–81) ^a^	55 (47–87) ^a^	86 (59–113) ^a,b^	77 (54–84) ^b^	<0.001

^1^ Differing intensity from 0.9 to 1.3 times the NWR threshold (TH). ^2^ Results of ANOVA on ranks (Friedman test). ^a,b,c^ Within a row, values with different superscript letters are significantly different (*p* < 0.05; Tukey Kramer test for pairwise comparisons). Relative Amplitude = ratio of RMS amplitude of the reflex component to the background EMG RMS amplitude. R1 = reflex activity appearing as the first event after stimulation. R2 = reflex activity appearing as the second event after stimulation.

**Table 3:** Median (interquartile range) values obtained during electromyographic recordings of the right deltoid muscle in 12 horses stimulated over the right lateral palmar digital nerve with 25-ms train-of-5 constant-current pulses (duration 1 ms) delivered by a computer-controlled stimulator at various stimulation intensities: nociceptive withdrawal reflex (NWR) threshold (TH) and tolerance (TO).

Variable	TH	TO	P ^1^
Reaction Score	2 (2–3)	5 (5–5)	
Relative Amplitude R1	7.3 (2.6–14.8)	66.5 (47.7–84.8)	<0.002
Relative Amplitude R2	19 (11.9–39)	78.2 (49.2–85)	<0.02
Latency R1 (ms)	44 (41–52)	39 (38–41)	<0.02
Latency R2 (ms)	81 (75–93)	70 (64–74)	<0.01
Duration R1 (ms)	29 (13–32)	58 (29–71)	<0.002
Duration R2 (ms)	66 (41–81)	69 (58–112)	<0.02

^1^ Results from Wilcoxon signed rank test. Relative Amplitude = ratio of RMS amplitude of the reflex component to the background EMG RMS amplitude. R1 = reflex activity appearing as the first event after stimulation. R2 = reflex activity appearing as the second event after stimulation.

## Data Availability

The data presented in this study are available on request from the corresponding author.
